# Efficacy of Ergonomic Interventions on Work-Related Musculoskeletal Pain: A Systematic Review and Meta-Analysis

**DOI:** 10.3390/jcm14093034

**Published:** 2025-04-28

**Authors:** Weiner Santos, Carmen Rojas, Rui Isidoro, Alejandro Lorente, Ana Dias, Gonzalo Mariscal, María Benlloch, Rafael Lorente

**Affiliations:** 1 International Doctoral School, University of Extremadura, 06006 Badajoz, Spain; weiner.santos1976@gmail.com; 2 Local Health Unit Litoral Alentejano (ULSLA), 7540-230 Santiago do Cacém, Portugal; 3 School of Industrial Engineering, University of Extremadura, 06006 Badajoz, Spain; cvrojas@unex.es; 4 Polytechnic Institute of Beja, Rua Pedro Soares, Campus do Instituto Politécnico de Beja, 7800-295 Beja, Portugal; rui.isidoro@ipbeja.pt (R.I.); ana.dias@ipbeja.pt (A.D.); 5 Life Quality Research Centre (LQRC-CIEQV), 2001-964 Santarém, Portugal; 6 Ankle and Foot Surgery Unit, Department of Traumatology and Orthopaedic Surgery, University Hospital Ramón y Cajal, 28034 Madrid, Spain; 7 Institute for Research on Musculoskeletal Disorders, Catholic University of Valencia San Vicente Mártir, 46001 Valencia, Spain; gonzalo.mariscal@mail.ucv.es; 8 Department of Basic Biomedical Sciences, Catholic University of Valencia San Vicente Mártir, 46001 Valencia, Spain; 9 Department of Orthopedic Surgery and Traumatology, University Hospital of Badajoz, 06010 Badajoz, Spain; rafael.lorentem@gmail.com

**Keywords:** ergonomics, work-related musculoskeletal disorders, pain

## Abstract

**Background**: Among the leading causes of work-related disability, musculoskeletal diseases (MSDs) profoundly affect productivity and quality of life. Workplace changes, equipment adjustments, and training courses, among other ergonomic interventions, seek to lower the frequency and degree of MSDs. This systematic review and meta-analysis evaluated whether ergonomic interventions help prevent and control MSDs in various workplace environments. **Methods**: A systematic search was conducted in PubMed, Scopus, Embase, Web of Science, and Cochrane Library to identify relevant studies. Inclusion criteria included randomized controlled trials (RCTs) that evaluated ergonomic interventions against conventional conditions. Effect sizes were computed using mean differences and pooled using a random-effects model in case of heterogeneity. A uniform Excel sheet was used for data extraction. Revman software (Cochrane Collaboration, Copenhagen, Denmark) was used for statistical analysis. **Results**: This meta-analysis included 24 RCTs with 4086 workers with different occupations. A meta-analysis of 10 included studies demonstrated lower pain intensity with a mean difference in VAS score between ergonomic interventions and the control group of −0.28 (95%CI: −0.43, −0.14, *p* = 0.0001). Also, there was a significant reduction in reported MSD-related pain in the lower back with ergonomic interventions with an OR 0.53 (95%CI: 0.40–0.70, *p* < 0.00001). Moreover, there were statistically significant results for ergonomic interventions in the upper back, ankles, wrists, and neck. In contrast, there were no significant improvements in the thighs, arms, knees, shoulders, and elbows. **Conclusions**: Our findings support implementing ergonomic strategies as a practical approach to reducing work-related MSDs. However, further research is needed to improve intervention design and long-term effectiveness.

## 1. Introduction

Musculoskeletal disorders (MSDs) are medical conditions that negatively impact muscles, tendons, ligaments, nerves, and other soft tissues, resulting in pain, discomfort, and disability. MSDs affect a wide range of professions and are primarily caused by repetitive motions, poor postures, over-lifting, and inadequate ergonomic workplaces. Musculoskeletal disorders have traditionally been linked to high-workload occupations, such as manufacturing, construction, and healthcare; however, they are increasingly prevalent in sedentary occupations due to inadequate ergonomic design and prolonged static postures [1,2].

Prolonged exposure to occupational risk factors increases the likelihood of developing MSDs, including lower back pain, carpal tunnel syndrome, tendinitis, and neck strain. Studies suggest poor workplace ergonomics can exacerbate these conditions, reducing work efficiency and job satisfaction [3]. The prevalence of MSDs varies by occupational sector. Studies indicate that in the European Union, over 30% and up to 80% of workers in physically demanding jobs report MSD-related complaints, with construction and healthcare among the most affected industries. Meanwhile, office workers report a rising incidence of MSDs, particularly in the neck, shoulders, and lower back, due to prolonged sitting and poor workstation design. The economic impact of MSDs is significant, with direct costs, such as medical expenses and rehabilitation, and indirect costs, such as absenteeism and reduced productivity. In the United States alone, MSDs account for one-third of all workplace injuries, costing employers an estimated USD 50 billion annually in compensation and lost productivity [2,4].

Furthermore, MSD-related injuries are one of the most common leading causes of disability worldwide, imposing a significant economic burden on employers and healthcare systems. In response to this growing concern, ergonomic interventions have become widely used to reduce MSD risk factors and improve workplace safety [4,5].

Ergonomic interventions include physical, ergonomic interventions that include tools and work environment adaptations, i.e., sit-stand desks, ergonomic chairs, and adjustable workstations. Cognitive ergonomics optimizes work processes for better mental workload management, attention, and decision-making. Training-based interventions in the form of employee education programs in workstation ergonomics, lifting, and posture have been widely applied. However, research indicates that while training may increase awareness, it results in minimal long-term behavior modification unless combined with physical and ergonomic interventions. Therefore, multifaceted ergonomic interventions, which integrate physical, cognitive, and training-based strategies, may offer a more comprehensive approach to MSD prevention [5,6,7].

Despite the widespread implementation of ergonomic interventions, their effectiveness remains debatable due to inconsistencies in study design, intervention methods, and outcome measurements. While some studies report significant reductions in MSD incidence following ergonomic adjustments, others find minimal or no long-term effects. This variability highlights critical gaps in the existing literature, including a lack of standardized methodologies, limited long-term follow-ups, and insufficient comparative analyses across different occupational settings.

Given these challenges, a systematic review and meta-analysis are essential to synthesize current evidence and provide a more comprehensive understanding of ergonomic interventions. Our study aims to determine the effects of ergonomic accommodations on pain relief across different body regions and work settings.

## 2. Materials and Methods

### 2.1. Study Design

This study is a systematic review and meta-analysis of randomized controlled trials (RCTs) assessing the efficacy of ergonomic interventions in preventing and managing MSDs in occupational settings. This study had a written protocol with review questions, a search strategy, inclusion/exclusion criteria, and a risk of bias assessment. We strictly adhered to the PRISMA guidelines to ensure methodological rigor and transparency [8]. The study protocol was registered on the Open Science Framework (OSF) under the following DOI: https://doi.org/10.17605/OSF.IO/QRE68 (accessed on 10 January 2025).

### 2.2. Eligibility Criteria

*Inclusion criteria:*

Our research question follows the PICOS strategy: Population (P): Workers in any occupational setting were exposed to ergonomic risk factors for MSDs; Intervention (I): Any ergonomic intervention, including physical, cognitive, training-based, or multifaceted interventions; Comparison (C): Studies comparing ergonomic interventions to no intervention, usual care, or other ergonomic interventions; Outcomes (O): Studies must report at least one of the following primary or secondary outcomes:

Primary Outcome: Reduction in musculoskeletal pain (measured through self-reported scales or clinical assessments).

Secondary Outcomes: Work-related disability, productivity loss, absenteeism, presenteeism, work engagement, or healthcare costs.

Language: Studies published in English.

*Exclusion Criteria:*

- Studies with non-occupational populations (e.g., general public, student populations).
- Studies lacking a control group.
- Studies with insufficient data for effect size estimation.
- Conference abstracts, case reports, and unpublished dissertations.

### 2.3. Literature Search

We performed a systematic literature search, using the following terms to search all trial registers and databases: ((“ergonomic” OR “ergonomic intervention”) AND (“work-related” OR “occupational”) AND (“musculoskeletal disorders” OR “MSDs”)).

A systematic search of PubMed, EMBASE, Scopus, Web of Science (WOS), and Cochrane Collaboration Library databases was conducted without applying date limits or language restrictions.

### 2.4. Study Selection

Two authors independently screened the titles and abstracts of the retrieved articles to identify potentially relevant studies. Following this initial screening, the full texts of the selected studies were assessed to determine their eligibility based on the inclusion criteria within two weeks. Any disagreements between the two reviewers were resolved through discussion, and if consensus was not reached, a third reviewer provided the final decision. Rayyan software v. 1.6.0 was used to screen the resulting articles. Endnote software v.21 was used to remove duplicates.

### 2.5. Data Extraction

Two authors independently extracted the required data from the final included studies using a tailored Excel sheet that suited our aims, extracting the following baseline characteristics obtained for each survey: study name, study design, region, type of ergonomic intervention, occupation, number of participants, age, and body mass index (BMI).

According to outcomes, we aim to focus on reported pain reduction after interventions and disability through extracting reported scores or clinical assessment.

### 2.6. Assessment of Risk of Bias

Two independent reviewers conducted the quality assessment of randomized controlled trials using the Cochrane Risk of Bias Tool 2, following a structured evaluation of key methodological criteria. These criteria included random sequence generation, allocation concealment, blinding of participants, personnel, and outcome assessors, handling of incomplete outcome data, and selective outcome reporting. Any disagreements between reviewers regarding the risk of bias assessment were resolved through discussion, and if consensus was not reached, a third reviewer provided the final decision.

### 2.7. Assessment of Results

The Review Manager 5.4 software package provided by the Cochrane Collaboration was used to perform the meta-analysis and generate forest plots. For dichotomous variables, odds ratios (ORs) with a 95% confidence interval (CI) were calculated. The standardized mean difference (SMD) and 95% CI were calculated for the continuous variables. Heterogeneity was checked using both the x^2^ and I^2^ tests. A fixed-effects model was employed if there was no statistical evidence of heterogeneity, and a random-effects model was adopted if notable heterogeneity was observed. In case of heterogeneity, sensitivity analyses were performed, and subgroup analysis was performed if needed.

### 2.8. Risk of Bias Across the Studies

We evaluated the potential for publication bias by analyzing a funnel plot in Review Manager 5.4.

### 2.9. Additional Analyses

A sensitivity analysis was performed in Revman by excluding one study. Also, if more than one study caused heterogeneity, we conducted a subgrouping analysis based on the occupation. To evaluate the quality of evidence and the strength of recommendations, the GRADE (Grading of Recommendations, Assessment, Development, and Evaluation) system was applied using GRADEpro. This framework assesses various factors, including study design, risk of bias, inconsistency, indirectness, imprecision, and an overall summary of findings.

## 3. Results

### 3.1. Study Selection

**Figure 1:**
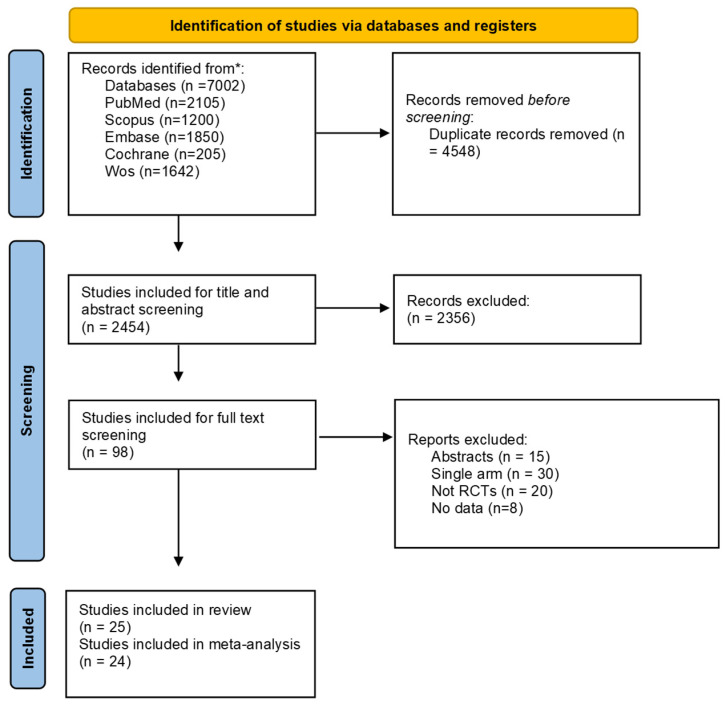
Study selection flow diagram. * Number of studies found in each database and total number in databases.

Our primary literature search, using our search strategy on different databases, yielded 7002 citations, of which 4548 duplicates were removed, 2454 articles were screened in the titles and abstracts screening step, ending up with 98 articles that were screened in the full-text screening stage. Finally, 25 RCTs were included in our study. After reviewing the references in the included articles, we found that no additional studies met our inclusion criteria. One study was included in the systematic review but excluded from the meta-analysis due to insufficient quantitative data for statistical pooling (Figure 1).

### 3.2. Study Characteristics

Table 1 presents the main characteristics of the studies included. Twenty-five studies included 2981 workers, most of whom were women. The mean age ranged from 27 to 60 years, and the mean BMI ranged from 21 to 29. Each group’s follow-up period ranged from 1 to 36 months. The ergonomic interventions included educational programs, training, and device-based interventions.

### 3.3. Risk of Bias Assessment

**Figure 2:**
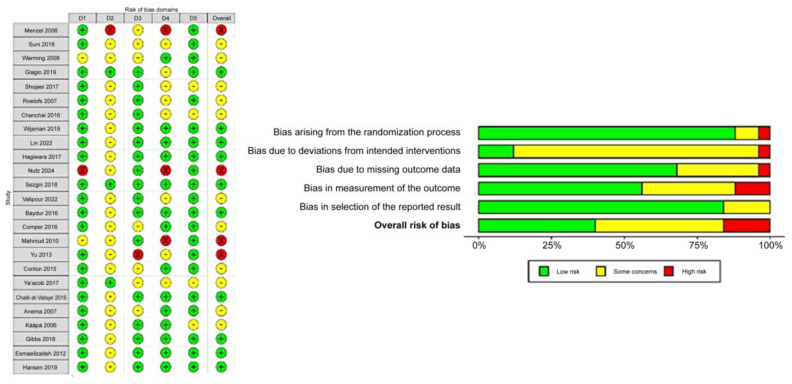
Risk of bias assessment of included studies [9,10,11,12,13,14,15,16,17,18,19,20,21,22,23,24,25,26,27,28,29,30,31,32,33].

Using the ROB2 tool, ten studies were identified with a low risk of bias [12,16,17,18,20,22,30,31,32,33]. Most of the studies were of moderate quality [10,11,13,14,15,21,23,26,27,28,29]. While four studies exhibited a high risk of bias [9,19,24,25], as illustrated in Figure 2.

### 3.4. Reported Pain Reduction in Different Body Parts

Our meta-analysis demonstrated a statistically significant reduction in reported musculoskeletal pain related to work among individuals who received ergonomic interventions compared to control groups. The pooled analysis of included studies yielded an odds ratio (OR) of 0.64 [95% CI: 0.56, 0.73]; *p* < 0.00001, indicating a significant reduction in pain through all body parts with ergonomic modifications. However, heterogeneity was moderate (I^2^ = 39%), suggesting variability in intervention efficacy across different studies (Supplementary Materials Figure S1).

### 3.5. Back Pain

**Figure 3:**
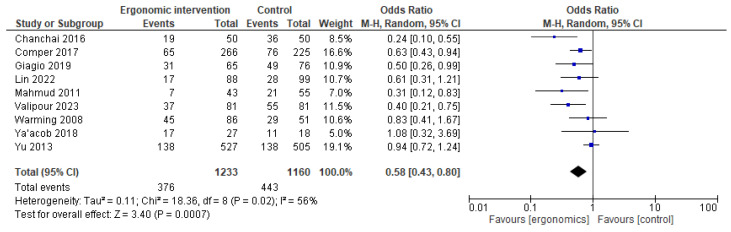
Graph showing reported pain reduction in the lower back at the last follow-up [11,12,15,17,21,23,24,25,27].

While analyzing the reported improvement in pain in the lower back, we found a significant improvement in ergonomics compared to controls, with a pooled OR of 0.58 [95% CI: 0.43, 0.80]; *p* = 0.0007, indicating a protective effect for ergonomics. Heterogeneity was moderate among the included studies with I^2^ = 56% (Figure 3).

A leave-one-out sensitivity analysis was performed by excluding Yu et al. [32], showing that heterogeneity was reduced (I^2^ = 24%) without changes in the context of the results. (Table 2).

**Figure 4:**
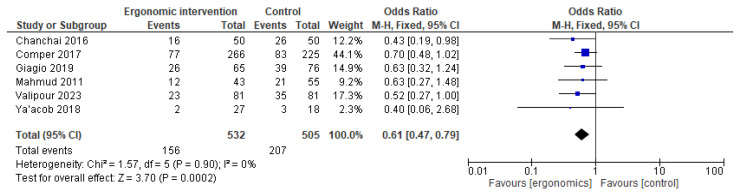
Graph showing reported pain reduction in the upper back at the last follow-up [12,15,21,23,24,27].

For upper back pain, ergonomics had a significant protective effect on reported pain, with an overall OR of 0.61 [95% CI: 0.47, 0.79]; *p* = 0.0002. There was no heterogeneity among the included studies, as shown in Figure 4.

### 3.6. Neck Pain

**Figure 5:**
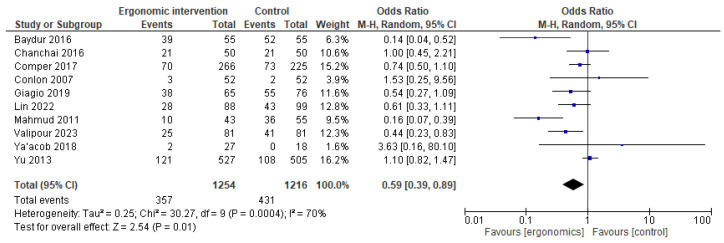
Graph showing reported pain reduction in the neck at the last follow-up [12,15,17,21,22,23,24,25,26,27].

The impact of ergonomic interventions on neck pain was interesting, with a protective effect. The pooled analysis showed a trend toward pain reduction. The OR was 0.59 [95% CI: 0.39, 0.89]; *p* = 0.01. Heterogeneity was high (I^2^ = 70%), indicating inconsistency among studies, as illustrated in Figure 5.

**Figure 6:**
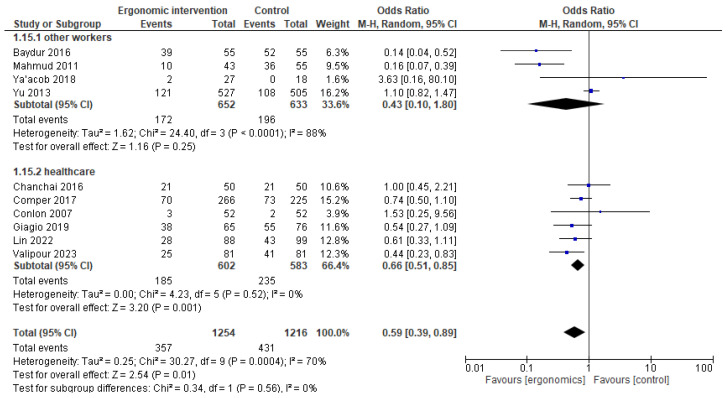
Graph showing a subgrouping analysis for neck pain reduction outcome based on population workplace to investigate heterogeneity among included studies [12,15,17,21,22,23,24,25,26,27].

A subgroup analysis was conducted based on the workplaces of healthcare workers or others. Interestingly, no heterogeneity among healthcare workers’ studies was found, with no change in the overall context of results, indicating a significantly better effect with ergonomics. In contrast, in the other group, heterogeneity was high, with I^2^ = 88%. A change in the results shows no difference between ergonomics and controls with *p* = 0.25, as illustrated in Figure 6.

### 3.7. Shoulder and Upper Limb Pain

**Figure 7:**
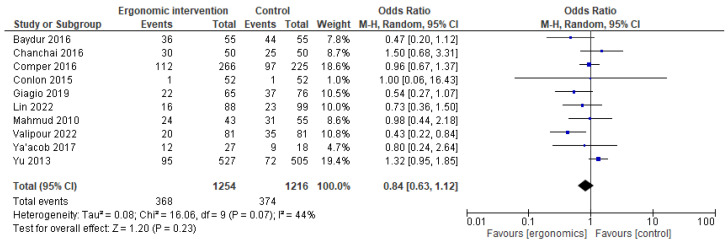
Graph showing reported pain reduction in shoulders at the last follow-up [12,15,17,21,22,23,24,25,26,27].

For shoulder pain, ergonomic interventions showed no statistical difference from controls (OR = 0.84 [95% CI: 0.63, 1.12]; *p* = 0.23), with moderate heterogeneity (I^2^ = 44%), as illustrated in Figure 7.

**Figure 8:**
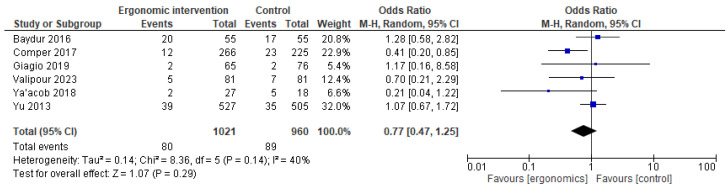
Graph showing reported pain reduction in the elbow at the last follow-up [12,21,22,23,25,27].

Similarly, ergonomic interventions did not show any significant reduction in reported elbow pain (OR = 0.77 [95% CI: 0.47, 1.25]; *p* = 0.29), with moderate heterogeneity (I^2^ = 40%), as shown in Figure 8.

**Figure 9:**
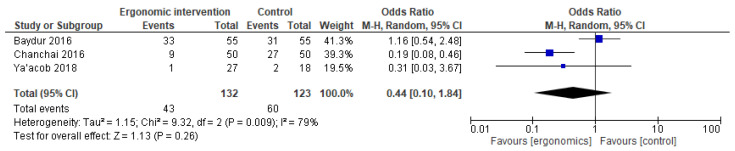
Graph showing reported pain reduction in the arms at the last follow-up [15,22,27].

Moreover, there was no significant difference in terms of arm pain (OR = 0.44 [95% CI: 0.10, 1.84]; *p* = 0.26), with moderate heterogeneity (I^2^ = 79%), as shown in Figure 9.

**Figure 10:**
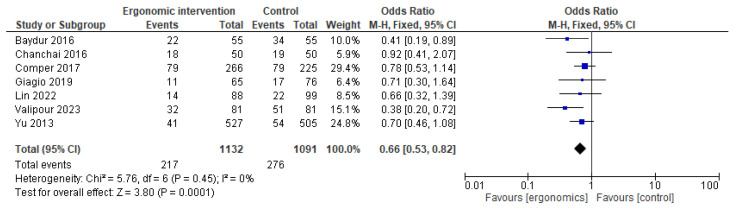
Graph showing reported pain reduction in wrists at the last follow-up [12,15,17,21,22,23,25].

In contrast to other parts in the upper limb, a statistically significant improvement with ergonomic interventions on reported wrist pain was found (OR = 0.66 [95% CI: 0.53, 0.82]; *p* = 0.26), with no heterogeneity (I^2^ = 0%), as illustrated in Figure 10.

Sensitivity analyses were performed by excluding one study in shoulder, elbow, and arm analyses to explore the source of heterogeneity. There was a significant reduction in heterogeneity in all of them with no changes in the context of the results, except for arm analysis, where the results were shifted to significance with *p* = 0.0002, as illustrated in Table 2.

### 3.8. Knee and Lower Limb Pain

**Figure 11:**
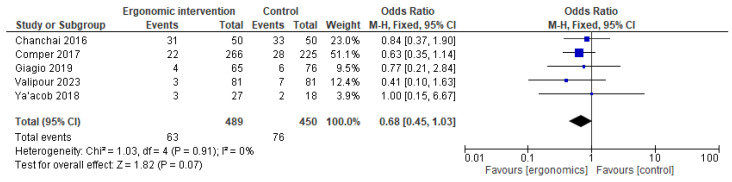
Graph showing reported pain reduction in thighs at the last follow-up [12,15,21,23,27].

**Figure 12:**
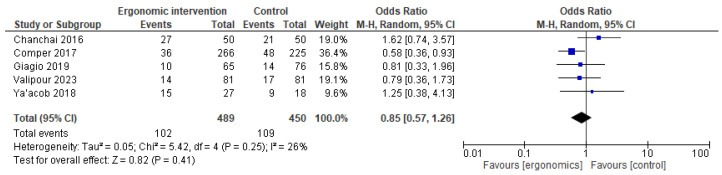
Graph showing reported pain reduction in knees at the last follow-up [12,15,21,23,27].

The effect of ergonomic interventions on thigh and knee pain was less pronounced. While some studies reported improvements, the pooled estimate showed no statistically significant reduction in thigh or knee pain compared to controls (OR = 0.68 [95% CI: 0.45, 1.03]; *p* = 0.07, I^2^ = 0%), (OR = 0.85 [95% CI: 0.57, 1.26]; *p* = 0.41, I^2^ = 26%), respectively, as shown in Figure 11 and Figure 12.

**Figure 13:**
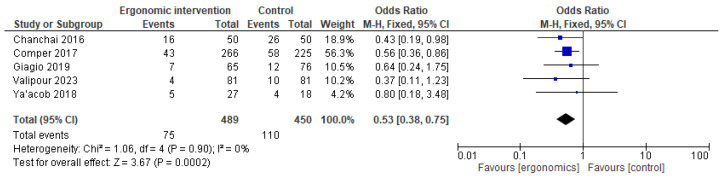
Graph showing reported pain reduction in ankles at the last follow-up [12,15,21,23,27].

However, ergonomics had a significant protective effect on ankle pain compared to controls (OR = 0.53 [95% CI: 0.38, 0.75]; *p* = 0.0002, I^2^ = 0%), as shown in Figure 13.

Interestingly, when sensitivity analysis was performed in knee analysis, by excluding Chanchai et al., heterogeneity was resolved, with a significant change in the overall results, showing that the ergonomics arm had a lower reported knee pain rate than the controls with *p* = 0.04, as shown in Table 2.

### 3.9. Pain Intensity

**Figure 14:**
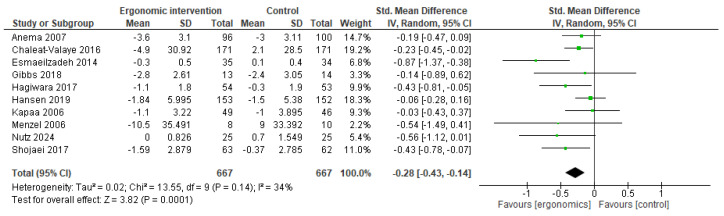
Graph showing the mean difference in pain intensity scores between ergonomic interventions and the control group at the last follow-up [9,13,18,19,28,29,30,31,32,33].

A meta-analysis of ten studies that reported different pain intensity scores was performed. Our analysis revealed that workers who used ergonomic interventions had lower pain scores compared to others, with SMD of −0.28 [95% CI, −0.43, −0.14; *p* = 0.0001). Also, a moderate level of heterogeneity was detected, with I^2^ = 34%, as illustrated in Figure 14.

A leave-one-out analysis was performed by excluding Esmaeilzadeh et al., and heterogeneity was resolved without any changes in the context of the overall results, as reported in Table 2.

### 3.10. Disability Scores

**Figure 15:**
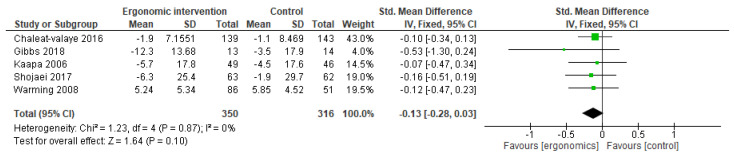
Graph showing the mean difference in disability scores between ergonomic interventions and the control group at the last follow-up [11,13,29,30,31].

Several studies included in the meta-analysis assessed functional disability scores following ergonomic interventions. The pooled analysis demonstrated no differences in disability scores between the ergonomic group and controls, with SMD of −0.13 [95% CI, −0.28, −0.03; *p* = 0.10). There was no heterogeneity among studies, with I^2^ = 0%, as illustrated in Figure 15.

### 3.11. Publication Bias and GRADE Assessment

**Figure 16:**
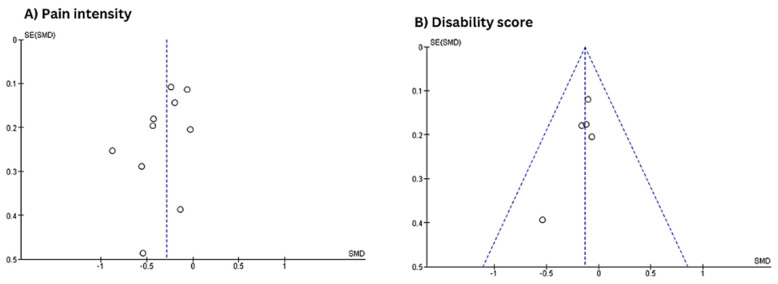
Funnel plot evaluating publication bias regarding pain intensity and disability scores.

The distribution of studies in the pain intensity funnel plot appears asymmetrical, suggesting potential publication bias or small-study effects. In contrast, the funnel plot for the disability score shows a relatively more symmetrical distribution around the central effect size, with points forming a triangular shape, as shown in Figure 16.

As mentioned in Table 3, the GRADE quality of evidence for these outcomes was rated as moderate, supporting the clinical relevance of ergonomic interventions in occupational settings.

## 4. Discussion

The existence of evidence for various ergonomic interventions to prevent and manage musculoskeletal disorders in different occupational environments is crucial. Our systematic review and meta-analysis show strong evidence for the effectiveness of transitional ergonomic interventions in decreasing work-related musculoskeletal pain. Based on evidence from 24 randomized controlled trials, we found significant decreases in pain, reported from different anatomical regions, among groups who received ergonomic interventions. Recent meta-analyses by Stucky et al. and Dogan et al. demonstrated that many surgeons and workers developed musculoskeletal pain, especially in the back, due to bad ergonomic management [34,35].

Our study results are broadly consistent with prior systematic reviews and meta-analyses evaluating the efficacy of ergonomic interventions. Van Niekerk et al. published a systematic review showing that the studies reviewed produced homogenous data, indicating that ergonomic modifications produced a statistically and clinically significant reduction in musculoskeletal pain post-intervention [36]. Manual techniques (mobilization and manipulation) were effective at relieving musculoskeletal pain, according to a meta-analysis investigating the contextual effects of physiotherapy interventions, contributing to the overall impacts of 81% and 88%, respectively [37].

Similarly, Russo et al. found that workplace interventions to improve musculoskeletal pain related to work had a good protective effect on workers in terms of pain and disability. However, we found that Russo et al. had issues with their reported data as they depended on the post-interventional scores without considering the change from baseline, with some errors in the extracted data [38]. Hoe et al. reported similar results regarding pain reduction in the neck, shoulder, and upper limb [5]. A systematic review and meta-analysis indicated that physiotherapist-led psychological interventions were superior to standard physiotherapy for chronic non-traumatic neck pain and acute whiplash. Marked enhancements in pain intensity, disability, and quality of life were noted during short-, medium-, and long-term evaluations [39].

Nevertheless, our study identified no statistically significant improvements in shoulder, elbow, or arm pain; Boocock et al. provided moderate evidence supporting the impact of keyboard and mouse design on health outcomes for visual display unit workers experiencing these conditions [40]. Interestingly, our analysis revealed a notable discrepancy regarding arm pain: while no significant effect was initially detected, removing the highest-weighted study led to a significant reduction in reported arm pain among participants in ergonomic intervention groups. Kennedy et al. also found moderate evidence supporting the benefits of arm supports, though workstation adjustments, ergonomic training, new chairs, and scheduled rest breaks demonstrated only limited effectiveness [41]. Van Eerd et al. [42] also reported moderate evidence favoring interventions such as mouse-use feedback, forearm supports, stress management training, and office workstation adjustments in alleviating musculoskeletal symptoms.

It is still unknown how ergonomic interventions affect lower limb pain, particularly in relation to knee and thigh discomfort, for which no appreciable changes were seen. Still, there was a notable protective effect for ankle discomfort. According to a recent meta-analysis, the type of intervention and the particular anatomical area targeted influence results concerning lower limb pain. Interventions aimed at correcting foot postures, including custom-made orthoses and targeted exercises, greatly reduced pain and disability according to a systematic review of physical treatments for chronic lower back pain linked with pronated feet [43]. These findings suggest that ergonomic interventions aimed at improving foot alignment and biomechanical support could play a role in mitigating ankle pain.

Recent rehabilitation strategies for neuropathic pain have shown that treatment that is a combination of cognitive behavioral treatment, mindfulness approaches, and structured physical treatment maximizes lower limb pain results to a considerable extent. Their efficacy is also dependent on the etiology of pain and treatment methods. This is to account for varying responses to knee and thigh pain, given that other variables aside from classic ergonomic treatment, such as degeneration of joints or systemic inflammatory disease, can impact the etiology of pain in these regions [44]. Furthermore, ergonomic accommodations in work settings have shown opposing effects on lower limb discomfort. Whereas evidence shows that workplace accommodations, such as anti-fatigue mats and sit-stand workstations, are associated with the relief of discomfort and fatiguability, their effects on knee and thigh pain are yet to be confirmed [45]. Interventions like supportive footwear and better surface ergonomics, which lower strain on the foot and ankle structures, could help to explain the protective effect noted for ankle pain.

Our findings suggest that ergonomic interventions eased pain but did not impact functional disability scores. This inconsistency is in keeping with more contemporary meta-analyses that suggest that relief of pain is not always linked to improvement in function, particularly in lower limb conditions. Alam et al. established that physical treatment of chronic low back pain with pronated feet eased the pain but inconsistently impacted functional disability, indicating that ergonomic adaptations can treat discomfort without easing total functional capacity [43]. One reason for a failure to generate improvement in function in response to pain relief is that ergonomic treatment addresses modifying extrinsic risk factors (e.g., posture, load distribution, support of work surface) without improving strength, mobility, or endurance per se. Disability in function is multifactorial, such that relief of pain, in conjunction with specific rehabilitation exercises, plus behavioral adjustment, is needed. Employers and personnel managers should implement ergonomic strategies tailored to different occupational environments. In physically demanding jobs such as construction, healthcare, and manufacturing, assistive lifting devices, anti-fatigue mats, and structured rest breaks can help reduce musculoskeletal strain, while training programs should focus on proper lifting techniques and load management. Office-based occupations would benefit from adjustable workstations, ergonomic chairs, optimized keyboard and mouse designs to minimize static postures and repetitive strain injuries, and scheduled movement breaks and stretching exercises [2,3,4]. For workers engaged in prolonged standing or walking, such as in retail and hospitality, supportive footwear and ergonomic flooring solutions can alleviate lower limb discomfort, while sit-stand workstations may provide additional benefits. High-cognitive and physically demanding professions, including surgery and factory work, require physical ergonomic modifications and cognitive workload management strategies, such as job rotation and stress management training, to enhance long-term musculoskeletal health [23,24,42].

The results support the need for combined intervention strategies that mitigate pain and enable active movement in conjunction with neuromuscular retraining and strengthening exercises to augment function more effectively. The results support that ergonomic interventions must be combined with organized physical treatment or exercise programs to augment function. Future studies must investigate the long-term effects of ergonomic adaptations in conjunction with movement-based treatment to ascertain if these interventions can mitigate pain and increase functional capacity.

### Limitations

Although our study provides robust evidence supporting ergonomic interventions, several limitations must be acknowledged. One major limitation is the heterogeneity in intervention types and workplace settings across the included studies. Differences in intervention protocols, occupational environments, and study methodologies may have influenced the results, despite statistical adjustments. Future studies should focus on standardizing ergonomic intervention protocols to improve comparability. Additionally, while ergonomic interventions clearly reduce pain, their long-term sustainability and worker adherence remain uncertain. More research is needed to evaluate how adherence rates influence the long-term benefits of ergonomic adaptations. Another limitation relates to the gender distribution in the included studies, as a majority of participants were female. Given that women generally report higher rates of musculoskeletal pain and may respond differently to ergonomic interventions due to physiological and occupational differences, future research should explore gender-specific ergonomic needs and intervention effectiveness. Measurement variability was another challenge, as studies reported pain intensity and disability using different scales, requiring the use of standardized mean differences in our meta-analysis. Furthermore, some studies described ergonomic interventions but did not report quantitative outcomes, limiting their inclusion. Future research should establish consistent outcome measures to enhance cross-study comparisons.

The need for further research is evident, particularly in addressing key gaps in ergonomic intervention effectiveness. Future studies should focus on determining which specific ergonomic interventions provide the most sustained benefits in different work environments. Research should also explore how gender differences influence responses to ergonomic modifications, given the higher representation of women in previous studies. Another important avenue of research involves identifying optimal combinations of ergonomic adjustments, exercise programs, and behavioral training to ensure long-term MSD prevention. Additionally, future studies should assess the broader impact of ergonomic interventions on workplace productivity, absenteeism, and healthcare costs. Longitudinal research incorporating objective biomechanical assessments and worker-reported outcomes is needed to evaluate the lifetime benefits of ergonomic adaptations. Comparative analyses between different types of ergonomic interventions will also be essential for developing personalized ergonomic strategies based on specific occupational risks.

## 5. Conclusions

This systematic review and meta-analysis supported that ergonomic interventions effectively reduce work-related musculoskeletal pain, particularly in the lower back, upper back, neck, wrist, and ankle. However, the findings indicate a small effect, raising concerns about the clinical significance of these findings. No significant improvements were found for shoulder, elbow, arm, thigh, or knee pain, and ergonomic interventions did not impact functional disability, suggesting they are not sufficient as standalone treatments. Since the effect size is relatively small, ergonomic interventions alone may not be sufficient. To maximize their benefits, they should be integrated with physical therapy, rehabilitation, and strength training. Future studies should investigate how well these interventions are maintained over time, how they can be tailored to different work environments, and their broader impact on productivity, absenteeism, and overall quality of life to better understand their practical value beyond statistical outcomes.

## Figures and Tables

**Table 1:** Baseline characteristics of included studies.

Study ID	Country	Type of Ergonomic (Education/Training/Device)	Study Arms	Age (Mean +SD)	M/F	Main Outcomes
Menzel 2006 [9]	USA	Education and training	Cognitive behavioral therapy (CBT)	40.3	5/27	The intervention group experienced a notable reduction in pain intensity, suggesting a substantial effect of the intervention. Nonetheless, levels of stress increased during the same period. Additionally, depressive symptoms emerged as a significant predictor, explaining a considerable proportion of the variance in work absenteeism due to back pain.
			Waitlist control group			
Suni 2018 [10]	Finland	Education and training	Combined neuromuscular exercise + back care counseling	45.1 (6.2)	53F	Combined-arm showed reduced intensity of LBP (*p* = 0.006) and pain interfering with work (*p* = 0.011) compared with the Control-arm. Work-related fear of pain was reduced in both the Combined- (*p* = 0.003) and Exercise-arm (*p* = 0.002). Physical activity-related fear was reduced only in the Exercise-arm (*p* = 0.008).
			Exercise alone (neuromuscular exercise)	47.2 (7.4)	57F	
			Counseling alone (back care counseling)	46.4 (6.4)	55F	
			Control (no intervention)	46.7 (7.2)	54F	
Warming 2008 [11]	Denmark	Education and training	Transfer technique education (TT) alone	-	-	The individual randomized intervention subgroup (transfer technique/physical training) significantly improved the LBP-disability (*p* = 0.001).
			Combined with physical fitness training (TTPT).	-	-	
			Control (nurses followed usual patient handling routine)	-	-	
Giagio 2019 [12]	Italy	Education, training, and devices	Preventive program (PP)	35.5 (10.9)	37/28	Among the surgeons included, physical activity was identified as the only modifiable risk factor for work-related musculoskeletal disorders (*p* = 0.05). The preventive program group showed significant improvements in general health at 3 and 6 months (*p* = 0.04), along with reductions in low back pain (*p* = 0.04) and analgesic use (*p* = 0.03).
			No-preventive program (NPP)	37.7 (12.1)	50/26	
Shojaei 2017 [13]	Iran	Education and training	Educational program and ergonomic posture training	-	52/11	Comparative analyses revealed a statistically significant decrease in the intensity of work-related low back pain from baseline in the group that participated in the multidisciplinary program (*p* < 0.001), while no meaningful changes were observed in the control groups. However, differences in disability scores between the intervention and control groups did not reach statistical significance (*p* = 0.07).
			Control (no intervention)	-	47/15	
Roelofs 2007 [14]	Netherlands	Device-based and education	Lumbar support + short course on healthy working methods.	41.8 (9.7)	3/180	Over the course of a year, participants who used lumbar support reported fewer days with low back pain compared to those who only received a brief educational intervention. However, this reduction in pain did not translate into a noticeable decrease in total sick leave days. The lumbar support group also showed slight but statistically significant improvements in pain intensity and functional capacity.
			Short course on healthy working methods only	41.5 (9.8)	5/172	
Chanchai 2016 [15]	Thailand	Education and training	Ergonomic training sessions	34.9 (9.5)	-	The study identified statistically significant variations in the prevalence of self-reported musculoskeletal disorders in the arm, upper back, and lower back regions when comparing data collected before and after the intervention. The results further indicated that the intervention had a measurable impact on certain psychosocial risk factors.
			Control (no intervention)	34.3 (7.3)	-	
Wijsman 2019 [16]	Netherlands	Device-based ergonomic intervention	AutoLap™ robotic camera holder was used during laparoscopic procedures.	60.7	-	Implementation of the AutoLap™ system leads to enhanced ergonomic conditions and postural benefits for the first assistant, while the surgeon’s ergonomic experience remains unchanged. Additionally, the use of a robotic camera holder is associated with a significant reduction in perceived workload.
			Standard laparoscopic procedures without a robotic camera holder.	59.6	-	
Lin 2022 [17]	China	Education and training	Participatory ergonomic (PE) training	27.12 (5.33)	31/57	Work ability scores showed improvement following the nine-month intervention. Compared to the control group, participants in the ergonomic program reported significantly fewer musculoskeletal issues in the neck and wrists/hands, along with a marginal increase in their work ability index, suggesting a positive—though limited—effect of the intervention.
			Single 45 min ergonomic education session (sham intervention).	28.25 (6.04)	31/68	
Hagiwara 2017 [18]	Japan	Device-based ergonomic intervention	(Wearable lumbar support—“spinal underwear”).	44.7 (10.0)	2/52	VAS and Somatosensory Amplification Scale scores as well as lumbar spinal ROM in the experimental group significantly decreased. Low back pain and neck pain in the experimental group significantly decreased.
			Waiting list control	44.7 (9.6)	1/52	
Nutz 2024 [19]	Germany	Device-based ergonomic intervention	Surgeons performed two surgeries of the same type—one without and one with the exoskeleton (Paexo Shoulder, Ottobock)	39 (7)	20/5	An upper body exoskeleton can significantly reduce the discomfort in the neck, shoulder, and back caused to surgeons by surgery.
			The first surgery was performed without the exoskeleton.			
Sezgin 2018 [20]	Turkey	Education and training	Ergonomic Risk Management Program (ERMP)	27 (5.21)	20/52	Six months post-intervention, nurses showed an average RULA score of 4.39 ± 1.49 during patient repositioning, suggesting the need for prompt ergonomic review. Pain levels, related medication use, and ergonomic risks significantly declined, while exercise frequency rose.
			Continued with regular ICU nursing duties without specific ergonomic training			
Valipour 2023 [21]	Iran	Education-based ergonomic intervention following the Theory of Planned Behavior (TPB).	Six weekly educational sessions (each ~1 h) covering ergonomic principles using TPB constructs.	32.2 (5.6)	18/63	At follow-up, % classified as high risk by ROSA dropped to 21% in the intervention group but rose to 65% in the control group. The intervention group reported significant symptom relief in the wrists/hands, lower back, neck, shoulders, and upper back (all *p* < 0.05), along with a notable reduction in the number of affected areas after three months.
			Control (No intervention)		14/67	
Baydur 2016 [22]	Turkey	Education and training	Training in office ergonomics and individual risk assessment.	36.0 (8.4)	47/69	Multivariate analysis showed a significantly lower likelihood of symptoms in the right neck and right wrist/hand in the intervention group (*p* < 0.05). Neck-related disability and symptom scores were also consistently lower over time compared to the control group (*p* < 0.05).
			Received an educational brochure at the end of the study.			
Comper 2017 [23]	Brazil	Education and training	Job rotation program	28.4 (7.8)	72/194	At 12 months, both groups reported increased sick leave hours due to musculoskeletal issues, with no significant difference between the job rotation group and controls. Secondary outcomes also showed no significant group differences (*p* > 0.05).
			Ergonomic training	32.5 (9.0)	30/195	
Mahmud 2011 [24]	Malaysia	Education and training	Office ergonomics training by NIOSH.	34.6 (10.4)	13/30	Workstation habits improved significantly and remained so at follow-up, particularly in keyboard, mouse, chair, and desk use. The greatest reduction in musculoskeletal disorders was in the neck (−42.2%, 95% CI −60.0 to −24.4). Significant improvements were also observed in the neck, right shoulder, upper and lower limbs, and lower back.
			Received a leaflet with ergonomic office tips	34.2 (8.4)	11/44	
Yu 2013 [25]	China	Education and training	Participatory training (POHSI model)	29.1 (7.3)	541/377	One year post-training, in the intervention group, MSD prevalence significantly declined in the lower extremities (*p* < 0.001) and in the wrist/fingers (*p* = 0.002). No significant changes were found in other body regions or in the control groups.
			Control_1 group was the control group in the intervention factory	28.9 (7.4)	516/391	
			Control_2 group was the control group in the control factory	28.3 (7.1)	914/740	
Conlon 2007 [26]	USA	Device-based ergonomic intervention	Alternative mouse	43.3 (10.8)	39/13	During the study, 42 participants developed new musculoskeletal disorders. Those using a forearm support board reported a significant reduction in right upper extremity discomfort. The alternative mouse showed a protective, but non-significant (*p* = 0.20) effect on right upper extremity disorders and neck/shoulder discomfort.
			Forearm support board	42.6 (10.3)	39/12	
			Alternative mouse + Forearm support board	44.4 (9.66)	36/15	
			Conventional mouse	41.2 (8.43)	35/17	
Ya’acob 2018 [27]	Malaysia	Education and training	MSS education and training and Kiken Yochi Training	34.94 (10.37)	27M	Postural assessment showed that 68.5% of participants had high-risk working postures for musculoskeletal symptoms. Tasks such as cultivation, manual weeding, and pineapple harvesting posed the greatest ergonomic risks. The most reported musculoskeletal symptoms were in the knees, lower back, and shoulders. Two months post-intervention, the intervention group showed a significant reduction in MSS prevalence in the ankles and feet.
			Received only MSS education and training	32.0 (9.07)	18M	
Anema 2007 [28]	Netherlands	Education and training	Workplace intervention, Yes	44.0 (8.6)	51/45	Median return-to-work time was shorter with workplace intervention (77 vs. 104 days, *p* = 0.02), which significantly improved return rates (*p* = 0.002). Graded activity negatively affected return to work (*p* < 0.001) and functional status. The combined intervention showed no effect.
			Workplace intervention, No	41.2 (10.7)	33/67	
			Graded activity, Yes	41.3 (9.2)	19/36	
			Graded activity, No	43.4 (8.3)	26/31	
Kääpä 2006 [29]	Finland	Education and training	Multidisciplinary rehabilitation	46 (7.9)	59F	No significant differences were found between the two treatment groups at post-rehabilitation, 6-, 12-, or 24-month follow-up. However, both intervention groups showed favorable effects in before-and-after comparisons, which were sustained at the 2-year follow-up.
			Individual physiotherapy	46.5 (7.0)	61F	
Gibbs 2018 [30]	USA	Education, training, and device-based ergonomic intervention	Behavioral counseling, sit-stand desk attachment, activity prompter, and pain self-management	52 (9)	2/11	Over six months, the intervention group sat 1.5 h/day less than controls (*p* < 0.001) and showed an average ODI reduction of 8 points (*p* = 0.001). At 6 months, ODI decreased by 50% in the intervention group vs. 14% in controls (*p* = 0.042). Although LBP reduction was not significant.
			No intervention (offered a 60 min lesson at the end of the study)	51 (13)	4/10	
Chaléat-Valaye 2016 [31]	France	Education and training	Education session + workplace training + home exercise program	47.1 (8.5)	39/132	At two-year follow-up, LBP recurrence with sick leave occurred in 24% of the intervention group and 21% of controls, with no significant difference (*p* = 0.516). The intervention significantly reduced fear-avoidance beliefs (*p* < 0.05) and improved muscle endurance (*p* < 0.05). It also reduced use of painkillers, medical visits, imaging, and physiotherapy.
			Usual care	47.3 (8.5)	38/133	
Hansen 2019 [32]	Denmark	Education and training	Occupational intervention + usual care	45.3 (10.1)	104/49	At 6 months, intention-to-treat analysis showed no significant difference in sick leave between groups (*p* = 0.42). Both groups improved significantly in pain (NRS), disability (RMDQ), fear-avoidance beliefs (FABQ), and physical HRQoL (SF-36), with no significant differences in secondary outcomes between them.
			Usual care only	45.7 (10.5)	102/50F	
Esmaeilzadeh 2014 [33]	Turkey	Education, training, and device-based ergonomic intervention	Ergonomic training and workstation adjustments	35.8 (6.5)	9/26	Over six months, the intervention group showed significant improvements in body posture (*p* < 0.001) and workstation layout (*p* = 0.002). WUEMSS intensity, duration, and frequency decreased significantly (all *p* < 0.01), alongside improvements in functional status and both physical (*p* < 0.001) and mental (*p* = 0.035) health-related quality of life. No significant change was observed in workday loss due to WUEMSS (*p* > 0.05).
			Control (no intervention)	35.0 (7.8)	11/23	

**ODI:** Oswestry Disability Index.

**Table 2:** Sensitivity analysis results for outcomes with heterogeneity.

Outcome	Excluded Study	Effect Size (95% CI)	*p*-Value	IÂ^2^ After Exclusion (%)
Lower back	Yu 2013 [32]	OR 0.53 (0.40, 0.70)	<0.00001	24%
Shoulder	Yu 2013 [32]	OR 0.76 (0.58, 1)	0.05	17%
Arms	Baydur 2016 [14]	OR 0.20 (0.08, 0.47)	0.0002	0
Elbow	Comper 2017 [26]	OR 1 (0.69, 1.44)	0.99	0
Knees	Chanchai 2016 [21]	OR 0.69 (0.49, 0.99)	0.04	0
Pain intensity	Esmaeilzadeh 2014 [15]	SMD −0.22 (−0.33, −0.11)	0.0001	0

**CI:** confidence interval; **OR:** odds ratio; **SMD:** standardized mean difference.

**Table 3:** Grade assessment of included outcomes.

**Certainty Assessment**							**№ of Patients**		**Effect**		**Certainty**	**Importance**
**№ of Studies**	**Study Design**	**Risk of Bias**	**Inconsistency**	**Indirectness**	**Imprecision**	**Other Considerations**	**Ergonomic Intervention**	**Control**	**Relative** **(95% CI)**	**Absolute** **(95% CI)**		
**Pain intensity**												
10	randomized trials	serious ^a^	not serious	not serious	not serious	none	667	667	-	SMD **0.28 SD lower** (0.43 lower to 0.14 lower)	⨁⨁⨁◯ Moderate ^a^	CRITICAL
**Disability score**												
5	randomized trials	serious ^a^	not serious	not serious	not serious	none	350	316	-	SMD **0.13 SD lower** (0.28 lower to 0.03 higher)	⨁⨁⨁◯ Moderate ^a^	IMPORTANT
**Reported pain reduced**												
11	randomized trials	serious ^a^	not serious	not serious	not serious	none	1409/6771 (20.8%)	1730/6405 (27.0%)	**OR 0.64** (0.56 to 0.73)	**79 fewer per 1000** (from 98 fewer to 57 fewer)	⨁⨁⨁◯ Moderate ^a^	IMPORTANT

^a.^ Most of studies rated as some concerns in the risk of bias; ⨁⨁⨁◯: Moderate certainty; **CI:** confidence interval; **OR:** odds ratio; **SMD:** standardized mean difference.

## Data Availability

Primary data for the systematic literature review are available in the referenced publications.

## Supplementary Materials

The following supporting information can be downloaded at: https://www.mdpi.com/article/10.3390/jcm14093034/s1, Figure S1: MSD reported pain reduction.

## Abbreviations

The following abbreviations are used in this manuscript:

MSDs	musculoskeletal diseases
BMI	body mass index
CI	confidence interval
GRADE	Grading of Recommendations, Assessment, Development, and Evaluation
LD	linear dichroism
ORs	odds ratios
PRISMA	Preferred Reporting Items for Systematic Reviews and Meta-Analyses
RCTs	randomized controlled trials
SMD	standardized mean difference
