# Supplementary figures and images for: Efficacy of Ergonomic Interventions on Work-Related Musculoskeletal Pain: A Systematic Review and Meta-Analysis
Source: J Clin Med. 2025 Apr 28;14(9):3034. doi: 10.3390/jcm14093034 (PMC12073017; doi:10.3390/jcm14093034)

## Supplementary Figure S1. MSD reported pain reduction

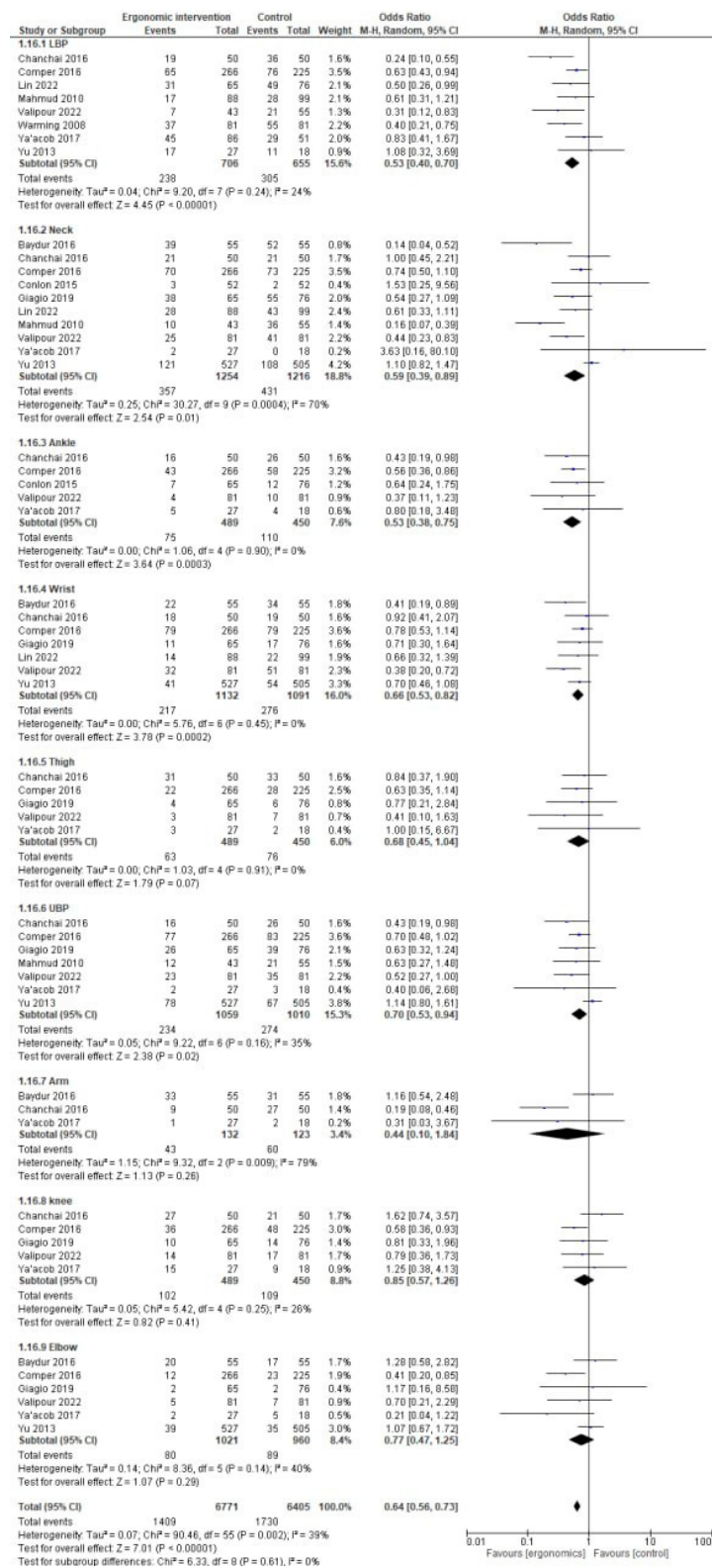

Supplement: Supplementary file 1 [file jcm-14-03034-s001.zip › jcm-3527182-supplementary.pdf]
